# Biochar Application Mitigates the Effect of Heat Stress on Rice (*Oryza sativa* L.) by Regulating the Root-Zone Environment

**DOI:** 10.3389/fpls.2021.711725

**Published:** 2021-08-05

**Authors:** Min Huang, Xiaohong Yin, Jiana Chen, Fangbo Cao

**Affiliations:** Crop and Environment Research Center, College of Agronomy, Hunan Agricultural University, Changsha, China

**Keywords:** biochar, heat stress, nitrogen utilization, rice, root zone

## Abstract

Coping with global warming by developing effective agricultural strategies is critical to global rice (*Oryza sativa* L.) production and food security. In 2020, we observed that the effect of heat stress on rice plants was mitigated by biochar application (40 g kg^−1^ soil) in a pot experiment with six consecutive days (6–11 days after transplanting) of daily mean temperatures beyond the critical high temperature (33°C) for tillering in rice. To further determine the eco-physiological processes underlying the effect of biochar on resistance to heat stress in rice plants, we compared root-zone soil properties as well as some plant growth and physiological traits related to nitrogen (N) utilization between rice plants grown with and without biochar in the pot experiment. The results showed that the application of biochar improved the root-zone environment of rice plants by reducing soil bulk density, increasing soil organic matter content, and altering soil bacterial community structure by increasing the ratio of Proteobacteria to Acidobacteria, for example. As a consequence, root morphology, architecture, and physiological traits, such as N assimilation and transport proteins, as well as shoot N uptake and utilization (e.g., photosystems I and II proteins), were improved or up-modulated, while the heat-shock and related proteins in roots and leaves were down-modulated in rice plants grown with biochar compared to those without biochar. These results not only expand our understanding of the basic eco-physiological mechanisms controlling increased heat-stress tolerance in rice plants by the application of biochar, but also imply that improving the root-zone environment by optimizing management practices is an effective strategy to mitigate heat stress effects on rice production.

## Introduction

Rice (*Oryza sativa* L.) feeds approximately 50% of the world’s human population, and rice yield must increase by about 1% annually to meet the growing food demand that will result from population growth and economic development (Normile, 2008). However, this task is not easy to achieve due to changes in socioeconomic and physical environments related to rice production (Peng et al., 2009). Among these changes, global climate change is expected to be a particularly serious challenge for global rice production in the near future (Wassmann et al., 2009).

Global warming, the phenomenon of rising average air temperatures near earth’s surface over the past 100–200 years, is a major aspect of climate change that threats rice production (Horie, 2019). Peng et al. (2004) reported that rice yield decreased by 10% when night temperatures during the growing season increased by 1°C, which is associated with global warming. Moreover, global warming has and will continue to increase the frequency and severity of extreme weather events, such as droughts and heatwaves (Stott, 2016; Perkins-Kirkpatrick and Lewis, 2020), which can partially or completely damage crop production (Lesk et al., 2016). Therefore, it is crucial to find effective agricultural strategies to cope with global warming in order to ensure global food security (Wassmann et al., 2009; Horie, 2019).

**Figure 1:**
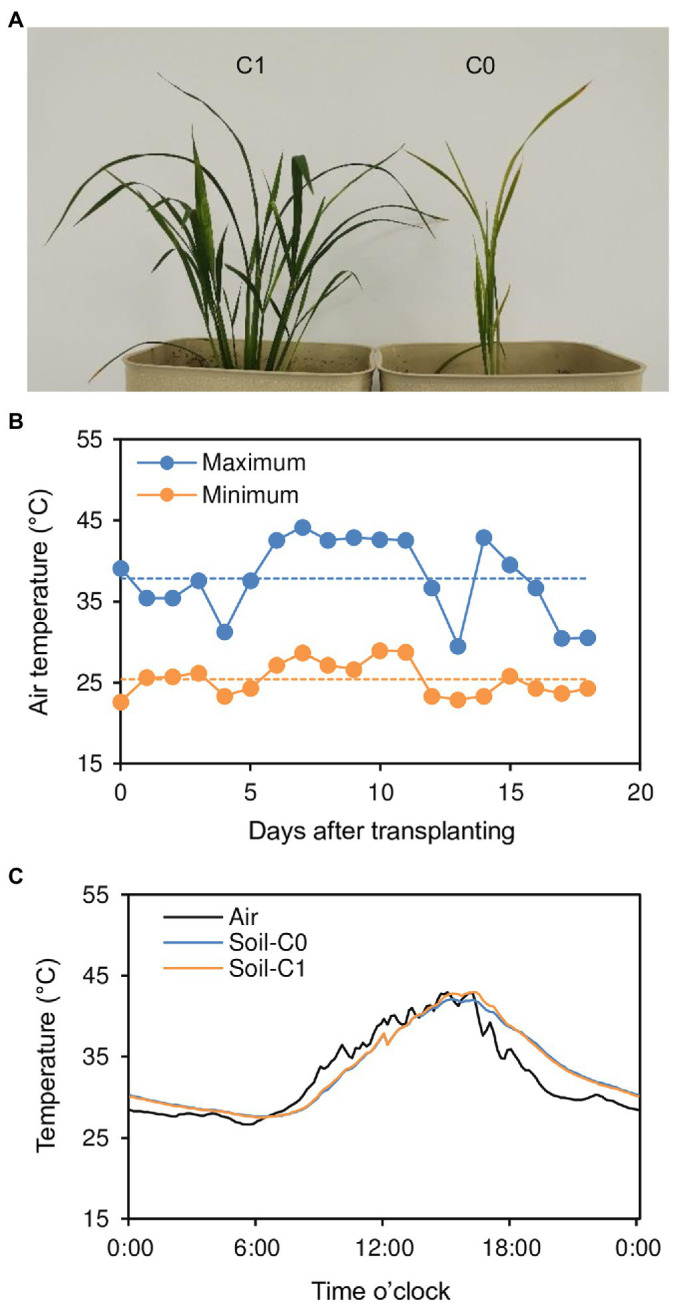
**(A)** Rice plants grown in pots with (C1) and without (C0) biochar application shown at 18 days after transplanting, **(B)** daily maximum and minimum air temperatures around rice plants during the experimental period, and **(C)** air and soil temperatures for the 24 h on day 9 after transplanting.

In 2020, we conducted a pot experiment to determine the effect of biochar application on rice growth (Figure 1A; see the Materials and Methods section for details). Because the pots were placed on concrete-covered ground exposed to the sun, high temperatures were induced during the experimental period. Average daily maximum and minimum air temperatures around the rice plants reached 37.9 and 25.4°C, respectively, during the period from transplanting to 18 days after transplanting (Figure 1B). Even more remarkably, there were six consecutive days (6–11 days after transplanting) with daily mean air temperatures (the average of daily maximum and minimum air temperatures) beyond the critical high air temperature (33°C) for tillering in rice (Krishnan et al., 2011). In addition, high soil temperatures also occurred during the experimental period. For example, maximum and minimum soil temperatures were 42.1 and 27.6°C, respectively, for the rice plants without biochar application (C0 treatment) and 43.0 and 27.5°C for the rice plants with biochar application (C1 treatment) in the nine-day period after transplanting, when the maximum and minimum air temperatures around the rice plants were 42.9 and 26.6°C, respectively (Figure 1C).

In general, high temperature (or heat stress) can adversely affect the rice growth by disturbing photosynthetic activity and respiration rate (Fahad et al., 2019). However, unexpectedly and interestingly, the high temperatures seriously limited the growth of rice plants in the C0 treatment but seemed to not affect the growth of those in the C1 treatment in our pot experiment (Figure 1A). This observation indicates that the effect of heat stress on rice can be mitigated by applying biochar to regulate the root-zone environment.

It has been well documented that biochar application can improve soil properties and consequently promote root growth and nitrogen (N) utilization in rice (Huang et al., 2013, 2014, 2018; Xiang et al., 2017). In addition, the previous studies have shown that improving N availability can mitigate the negative effects of heat stress on several crops, such as rice, maize, and potato (Tawfik et al., 1996; Ordóñez et al., 2015; Liu et al., 2019). Heckathorn et al. (1996) found that N availability is closely related to patterns of accumulation of heat-shock proteins in plants, which are generally induced by heat stress and play an important role in the development of tolerance to heat stress (Parsell et al., 1993). Therefore, we hypothesized that the increased resistance to heat stress in rice plants in the C1 treatment compared to those in the C0 treatment is partly attributable to improved N utilization.

In this study, root-zone soil properties as well as some plant growth and physiological traits related to N utilization were compared between the C0 and C1 treatments. The objective of this study was to determine the eco-physiological processes underlying resistance to heat stress in rice by altering the root-zone environment by adding biochar to the soil.

## Materials and Methods

### Plants and Treatments

The pot experiment was conducted at the Crop and Environment Center, Hunan Agricultural University, Changsha, China, in 2020. “Longliangyouhuazhan,” a high-yielding hybrid rice variety, was grown in pots without (C0) and with biochar application (C1). Each treatment consisted of six pots (replications). The pots were placed on concrete-covered ground exposed to the sun, causing high temperatures and heat stress for rice plants (see the Introduction section for details).

The soil used in the experiment was taken from the upper 20 cm layer of a rice field at the Experimental Farm of Hunan Agricultural University. The soil was a Fluvisol (FAO classification) with clay loam texture, pH = 5.99, organic matter = 22.7 g kg^−1^, total *N* = 1.32 g kg^−1^, and available *N* = 114 mg kg^−1^. The soil was air-dried, sieved, mixed, and then used to fill plastic pots (25-cm height, 20-cm length, and 15-cm width) with a weight of 5 kg pot^−1^ and a depth of ~20 cm.

The tested biochar was a rapeseed straw biochar with specific surface area = 3.02 m^2^ g^−1^, average bore size = 2.14 nm, pH = 10.8, total *C* = 440 g kg^−1^, and total *N* = 10.7 g kg^−1^. The application rate of the biochar was 200 g pot^−1^ (or 40 g kg^−1^ soil) for the C1 treatment. This biochar application rate was selected according to a preliminary screening, which indicated that rice plants grown under a biochar rate of 40 g kg^−1^ soil performed better than those under a recommended rate (20 g kg^−1^ soil) by Zhang et al. (2013). The biochar was applied 1 day before transplanting and was uniformly incorporated into the entire soil layer.

Pre-germinated seeds were sown in a seedling tray on 14 May. Twenty-day-old seedlings were transplanted into the pots with one hill per pot and one seedling per hill. Fertilizers used were urea for N, single superphosphate for phosphorus (P), and potassium chloride for potassium (K) at doses of 0.35 g N pot^−1^, 0.10 g P_2_O_5_ pot^−1^, and 0.10 g K_2_O pot^−1^, respectively. The N fertilizer was split-applied: 0.25 g N pot^−1^ 1 day before transplanting and 0.10 g N pot^−1^ 7 days after transplanting. The P and K fertilizers were both applied 1 day before transplanting. A water depth of ~3 cm above the soil surface was maintained during the experimental period.

### Soil Sampling and Measurements

Soils were sampled from three pots (replications) selected at random 18 days after transplanting to determine bulk density, organic matter content, available N content, and the bacterial community. All the soil samples were taken from the upper 10 cm layer where the rice roots were mainly distributed on the sampling day.

The bulk soil density was determined using the core method (Blake and Hartge, 1986). The organic matter content was measured by the potassium dichromate method (Walkley, 1947), and the available N content was assayed by the alkali-hydrolysis and diffusion method (Cornfield, 1960).

The bacterial community was investigated by analysis of the 16S rRNA gene sequence, which was performed by Novagene (Beijing, China). Quality-filtered and non-chimeric sequences were clustered to generate operational taxonomic units (OTUs) at a similarity level of 97% using UPARSE, v7.0.1090 (Edgar, 2013). Taxonomic information for the OTUs was annotated against the GreenGene database (DeSantis et al., 2006) using the RDP classifier algorithm, v2.2 (Wang et al., 2007). Bacterial community diversity and richness indices, including observed species, and the Shannon, Simpson, Chao1, Good coverage, and abundance-based coverage estimators, and the phylogeny-based metrics (PD whole tree) were calculated using QIIME, v1.9.1 (Caporaso et al., 2010). The relative abundance of each phylum was calculated by dividing the number of OTUs affiliated with a phylum by the total number of OTUs.

### Plant Sampling and Measurements

On the day of soil sampling, roots and the uppermost fully expanded leaves were sampled from the three soil-sampled pots for proteomic analysis, and whole rice plants were sampled from the other three pots to measure root traits, including root length, diameter, surface area, and dry weight, and shoot traits, including tiller number per plant, leaf area per plant, specific leaf weight (SLW), leaf N content (LNC), shoot dry weight, and shoot N uptake.

The proteomic analysis was carried out by PTM BioLab Co., Ltd. (Hangzhou, China), using tandem mass tag (TMT) coupled to liquid chromatography-mass spectrometry/mass spectrometry (LC-MS/MS) in accordance with the following procedures:

#### Protein Extraction

The sample was ground into fine powder in liquid N_2_ and transferred to a 5 mL centrifuge tube. A four-fold volume of lysis buffer (including 10 mm dithiothreitol and 1% protease inhibitor cocktail) was added to each sample and then sonicated three times on ice using a high-intensity ultrasonic processor (Scientz, Ningbo, China). An equal volume of Tris-saturated phenol (pH 8.0) was added, and then, the mixture was vortexed for 5 min. After centrifugation (4°C, 10 min, 5,500 *g*), the upper phenol phase was transferred to a clean centrifuge tube. Proteins were precipitated by adding five volumes of 0.1 M ammonium acetate-saturated methanol and incubated at −20°C overnight. After centrifugation at 4°C for 10 min, the supernatant was discarded. The remaining precipitate was washed once with ice-cold methanol, followed by washing three times with ice-cold acetone. The protein was redissolved in 8 M urea, and the protein concentration was determined with the bicinchoninic acid kit (Thermo Fisher Scientific, Rockford, IL, United States) according to the manufacturer’s instructions.

#### Trypsin Digestion

The protein solution was reduced with 5 mm dithiothreitol at 56°C for 30 min and alkylated with 11 mm iodoacetamide at room temperature in darkness for 15 min. The protein sample was then diluted by adding 200 mm triethylammonium bicarbonate (TEAB) to insure the urea concentration <2 M. Finally, trypsin was added at 1:50 trypsin-to-protein mass ratio for the first digestion overnight and at 1:100 trypsin-to-protein mass ratio for a second 4-h digestion.

#### TMT Labeling

After trypsin digestion, the peptide was desalted by Strata X C18 SPE column (Phenomenex, Torrance, CA, United States) and dried by vacuum centrifuging. Peptides were reconstituted in 0.5 M TEAB and labeled by using TMT kit (Thermo Fisher Scientific, Rockford, IL, United States) according to the manufacturer’s protocol. Briefly, one unit of TMT reagent was thawed and reconstituted in acetonitrile. The peptide mixtures were then incubated for 2 h at room temperature and pooled, desalted, and dried by vacuum centrifuging.

#### High-Performance Liquid Chromatography Fractionation

The tryptic peptides were fractionated into fractions by high pH reverse-phase high-performance liquid chromatography (HPLC) using Agilent 300 Extend C18 column (5 μm particle size, 4.6 mm internal diameter, and 250 mm length). Briefly, peptides were first separated with a gradient of 8–32% acetonitrile (pH 9.0) over 60 min into 60 fractions. Then, the peptides were combined into nine fractions and dried by vacuum centrifuging.

#### LC-MS/MS Analysis

The tryptic peptides were dissolved in solvent A (0.1% formic acid and 2% acetonitrile), directly loaded onto a home-made reversed-phase analytical column (15 cm length and 75 μm internal diameter). The gradient was comprised of an increase from 7 to 26% solvent B (0.1% formic acid and 90% acetonitrile) over 26 min, 26–38% in 8 min and climbing to 80% in 3 min then holding at 80% for the last 3 min, all at a constant flow rate of 500 nl/min on an EASY-nLC 1,000 ultra-performance liquid chromatography (UPLC) system (Thermo Fisher Scientific, Waltham, MA, United States). The peptides were subjected to a N solution index source followed by MS/MS in Q ExactiveTM Plus mass spectrometer (Thermo Fisher Scientific, Bremen, Germany) coupled online to the UPLC. The electrospray voltage applied was 2.2 kV. The m/z scan range was 400–1,500 for full scan, and intact peptides were detected in the Orbitrap at a resolution of 120,000. Peptides were then selected for MS/MS using a normalized collision energy setting at 28, and the fragments were detected in the Orbitrap at a resolution of 15,000. Automatic gain control was set at 5E4. Fixed first mass was set as 100 m/z.

#### Database Search

The resulting MS/MS data were processed using Maxquant search engine (v.1.5.2.8). Tandem mass spectra were searched against UniProt *O. sativa*_indica database (37,383 sequences) concatenated with reverse decoy database. Trypsin/P was specified as cleavage enzyme allowing up to two missing cleavages. The mass tolerance for precursor ions was set at 10 ppm in the first search and at 5 ppm in the main search, and the mass tolerance for fragment ions was set at 0.02 Da. Carbamidomethyl-modified cysteine residues were specified as a fixed modification, and oxidation of methionine was specified as a variable modification. The quantitative method is set to TMT-6plex. Both the false discovery rate for the identification of protein and propensity score matching were adjusted to <1%.

The root length, diameter, and surface area were determined using a WinRHIZO root analyzer system (Regent Instruments Inc., Quebec, Canada). Leaf area was measured with a LI-3000C leaf area meter (Li-Cor Inc., Lincoln, United States). The N content in the shoot (leaf and stem) was assayed using a Skalar SAN Plus segmented flow analyzer (Skalar Inc., Breda, Netherlands). The root and shoot (leaf and stem) dry weights were determined after oven-drying at 70°C to a constant weight. The SLW was calculated by dividing the leaf dry weight by the leaf area. The shoot N uptake was calculated by multiplying the shoot dry weight by the shoot N content.

### Data Analysis

For statistical analysis of proteomics data, the log2-fold change of mean value of protein quantity in the C1 compared to the C0 treatment was calculated for each quantifiable protein and then subjected to two-tailed Fisher’s exact test. The proteins with values of *p* < 0.05 and fold changes >1.3 or <1/1.3 were considered to be differentially modulated. The differentially modulated proteins were annotated based on the Gene Ontology (GO) and the Kyoto Encyclopedia of Genes and Genomes pathway databases. For analysis of other data, differences between the C1 and C0 treatments were evaluated by Student’s *t-*test, with significance levels of 0.05, 0.01, and 0.001.

## Results

### Root-Zone Soil Properties

**Figure 2:**
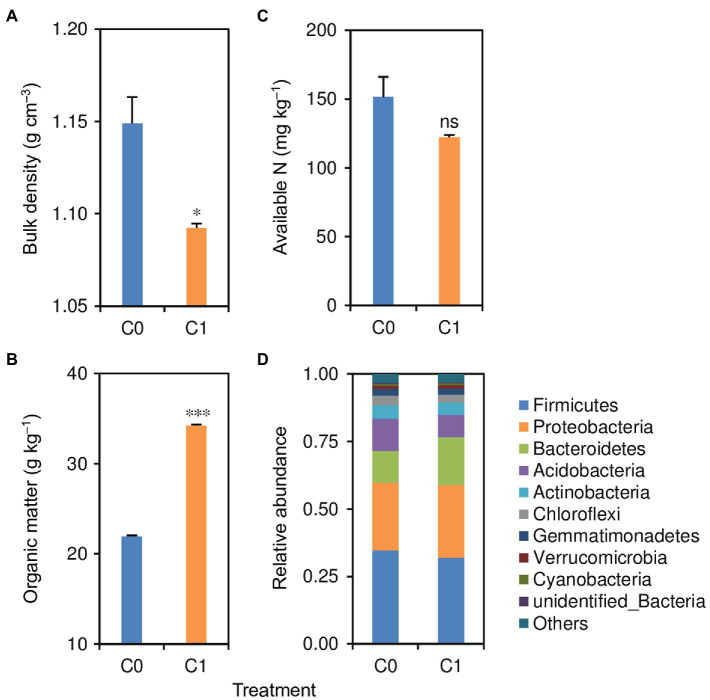
**(A)** Soil bulk density, **(B)** organic matter content, **(C)** available N content, and **(D)** relative abundance of bacterial phyla in treatments with (C1) and without (C0) biochar application. ^*^ and ^***^ indicate that the means of the rice plants in C1 treatment are significantly different from those in the C0 treatment at the 0.05 and 0.001 levels, respectively. “ns” indicates that the mean of the rice plants in C1 treatment is not significantly different from that in the C0 treatment at the 0.05 level.

Biochar application significantly affected the soil bulk density and organic matter content but did not significantly affect soil available N content (Figures 2A–C). The C1 treatment had 5% lower soil bulk density but 56% higher soil organic matter content than did the C0 treatment.

Biochar application had no significant effects on soil bacterial diversity and richness (Table 1). However, the structure of the soil bacterial community was changed by the application of biochar (Figure 2D). Compared with the C0 treatment, the C1 treatment had 8–31% lower relative abundances of Firmicutes, Acidobacteria, Actinobacteria, Chloroflexi, and Gemmatimonadetes but 4–49% higher relative abundances of Proteobacteria, Bacteroidetes, Verrucomicrobia, and Cyanobacteria.

**Table 1:** Soil bacterial community diversity and richness for rice plants grown with biochar application (C1) and those grown without biochar application (C0).

Index	C0	C1	Value of *p*
Observed species	2,824	2,770	0.247
Shannon	9.41	9.31	0.248
Simpson	0.995	0.995	1.000
Chao1	2,970	3,012	0.508
Good coverage	0.994	0.992	0.703
Abundance-based coverage estimators (ACE)	3,004	3,026	0.184
Phylogeny-based metrics (PD whole tree)	199	204	0.195

### Plant Growth Traits

**Figure 3:**
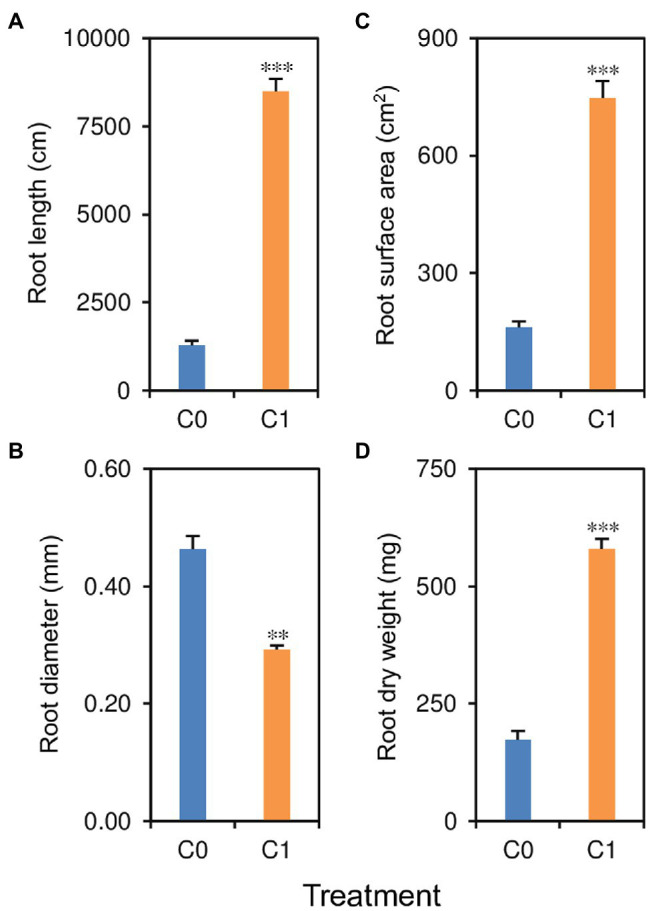
**(A)** Root length, **(B)** root diameter, **(C)** root surface area, and **(D)** root dry weight in rice plants grown with (C1) and without (C0) biochar application. ^**^ and ^***^ indicate that the means of the rice plants in C1 treatment are significantly different from those in the C0 treatment at the 0.01 and 0.001 levels, respectively.

Biochar application had significant effects on root length, diameter, surface area, and dry weight (Figures 3A–D). Rice plants in the C1 treatment had 235–561% greater root length, surface area, and dry weight but 37% lower root diameter compared to those in the C0 treatment.

**Figure 4:**
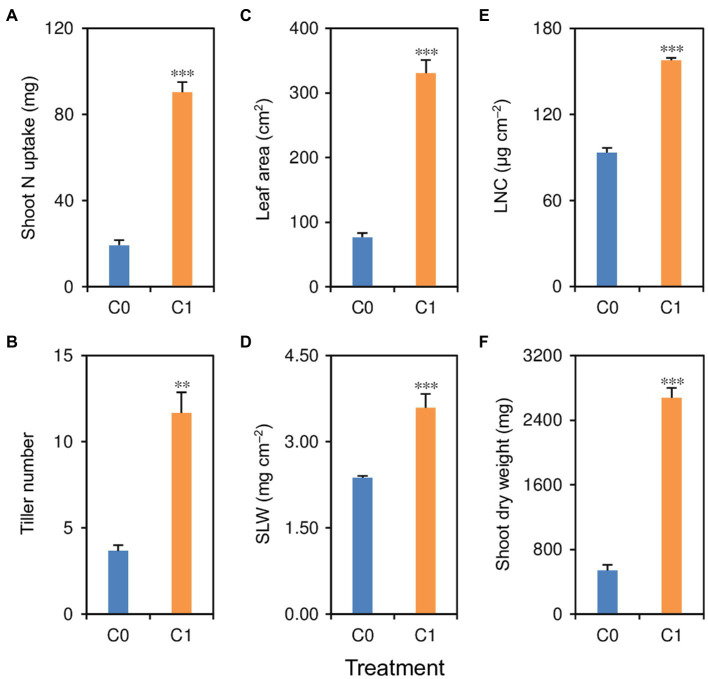
**(A)** Shoot N uptake, **(B)** tiller number per plant, **(C)** leaf area per plant, **(D)** specific leaf weight, **(E)** leaf N content, and **(F)** shoot dry weight in rice plants grown with (C1) and without (C0) biochar application. ^**^ and ^***^ indicate that the means of the rice plants in C1 treatment are significantly different from those in the C0 treatment at the 0.01 and 0.001 levels, respectively.

Shoot traits, including shoot N uptake, tiller number, leaf area, SLW, LNC, and shoot dry weight, were significantly affected by biochar application (Figures 4A–F). Compared with rice plants in the C0 treatment, rice plants in the C1 treatment had 51–396% higher shoot N uptake, tiller number, leaf area, SLW, LNC, and shoot dry weight.

### Plant Physiological Traits

**Figure 5:**
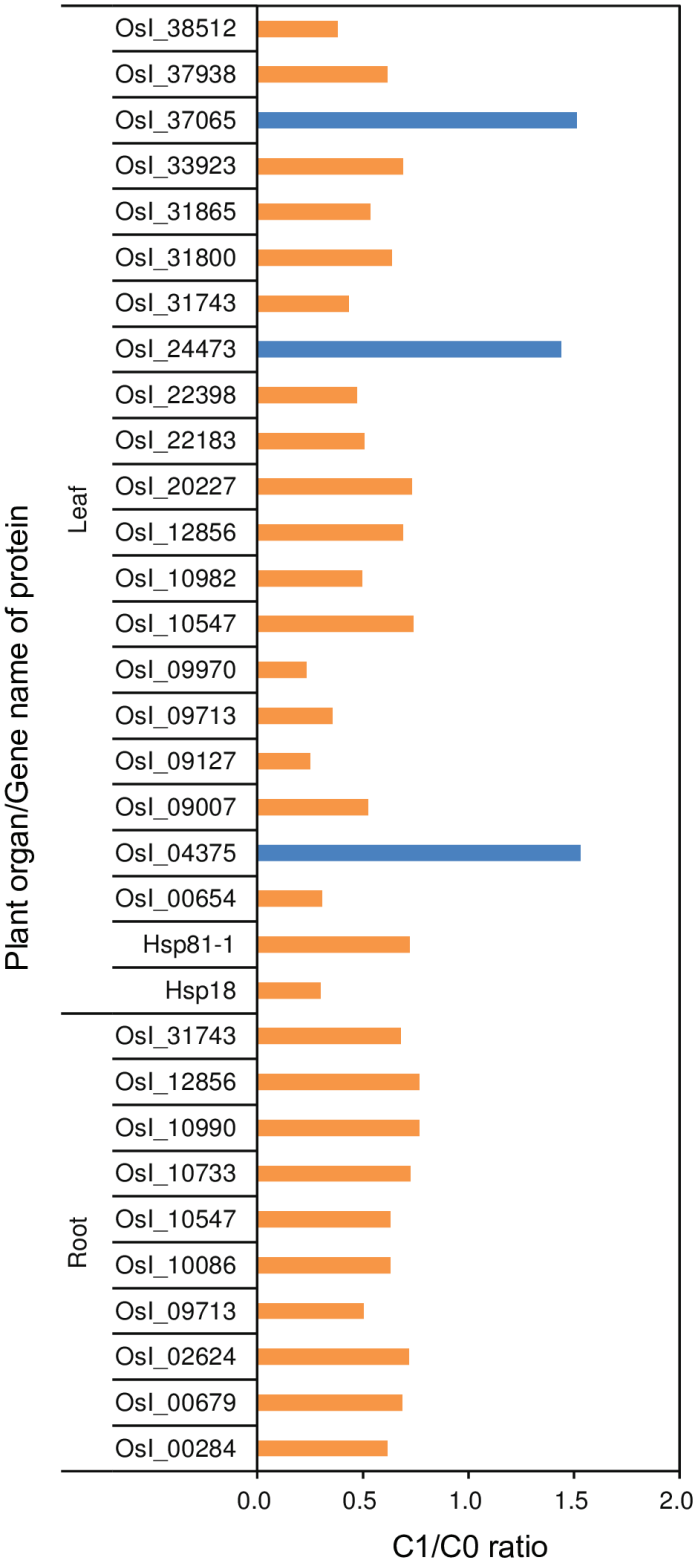
Fold changes of differentially modulated heat-shock and related proteins in roots and leaves of the rice plants grown with biochar application (C1) compared to plants grown without biochar application (C0). Orange and blue bars represent the down- and up-modulated proteins in the C1 treatment compared to the C0 treatment, respectively, respectively.

Total number of proteins identified in roots and leaves were 6,670 and 5,326, respectively, of which 4,975 and 4,013, respectively, were quantifiable (data not shown). Biochar application significantly modulated proteins in both roots and leaves. A total of 835 and 2084 differentially modulated proteins were detected in roots and leaves of C1 compared to C0 rice plants, respectively (data not shown). In particular, 10 and 22 differentially modulated heat-shock or related proteins were identified in roots and leaves, respectively (Figure 5). All 10 of the identified differentially modulated heat-shock or related proteins in roots and 20 of the 22 identified differentially modulated heat-shock or related proteins in leaves were down-modulated in rice plants in the C1 treatment compared to those in the C0 treatment with fold changes of 0.234–0.769.

**Figure 6:**
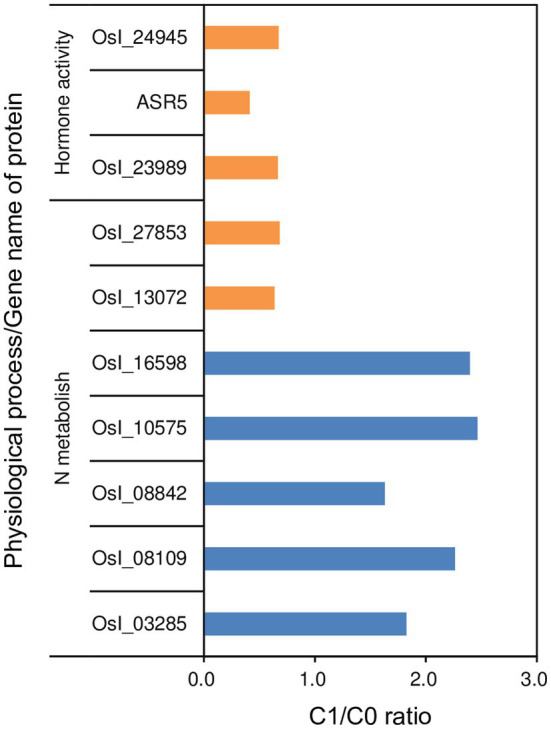
Fold changes in differentially modulated proteins related to N metabolism and hormone activity in roots of rice plants grown with biochar application (C1) compared to plants grown without biochar application (C0). Orange and blue bars represent the down- and up-modulated proteins in the C1 treatment compared to the C0 treatment, respectively.

**Figure 7:**
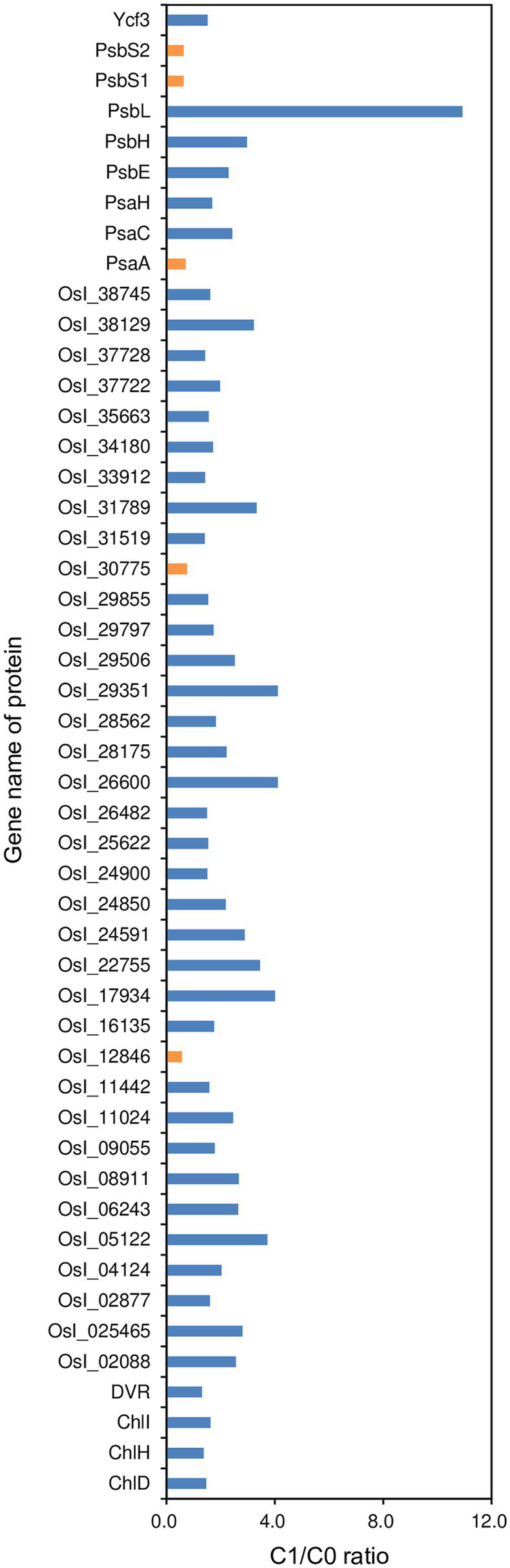
Fold changes in differentially modulated proteins related to photosystems I and II in leaves of rice plants grown with biochar application (C1) compared to plants grown without biochar application (C0). Orange and blue bars represent the down- and up-modulated proteins in the C1 treatment compared to the C0 treatment, respectively.

In addition, we identified seven and three differentially modulated proteins in roots that are related to N metabolism and hormone activity, respectively (Figure 6). Five out of the seven differentially modulated proteins related to N metabolism were up-modulated with fold changes of 1.63–2.47, while the remaining two differentially modulated proteins related to N metabolism and all three differentially modulated proteins related to hormone activity were down-modulated with fold changes of 0.41–0.68 in rice plants in the C1 treatment compared to those in the C0 treatment. The five up-modulated proteins related to N metabolism were glutamine synthetase (OsI_08842 and OsI_10575), glutamine amidotransferase type-2 domain-containing protein (OsI_03285), and two ammonium transporters (OsI_08109 and OsI_16598), while the two down-modulated proteins related to N metabolism were both nitrate transporters (OsI_13072 and OsI_27853). The three down-modulated proteins related to hormone activity were abscisic stress-ripening protein 5 (ASR5), auxin efflux carrier component (OsI_23989), and ethylene-insensitive 2 (OsI_24945). We identified 49 differentially modulated proteins in leaves that are related to photosystems I and II, of which 44 differentially modulated proteins were up-modulated in rice plants in the C1 treatment compared to those in the C0 treatment with fold changes of 1.314–10.931 (Figure 7).

## Discussion

Heat-shock proteins are generally up-modulated in response to high temperatures (Parsell et al., 1993). In this study, the down-modulation of several heat-shock or related proteins in C1 compared to C0 rice plants under high temperature conditions indicates that the effect of heat stress was mitigated in the C1 treatment. This finding is consistent with the performance of plant growth traits and, again, demonstrates that the effect of heat stress on rice can be mitigated by regulating the root-zone environment through biochar application.

In the present study, we found that biochar application improved the root-zone soil physical and chemical properties, including reduced bulk density and increased organic matter content. It is well known that reduced soil bulk density can reduce resistance to root penetration and increase root development (Lijima et al., 1991). In this study, we found that root length, surface area, and dry weight were consistently higher in rice plants in the C1 treatment with lower soil bulk density compared to those in the C0 treatment. The improvements in root length, surface area, and dry weight in the C1 treatment might also be partially attributed to the increased organic matter content, which plays an important role in maintaining soil quality because it has a positive effect on a wide range of soil properties, such as reducing soil compaction (Soane, 1990).

This study also showed that biochar application altered the structure of the soil bacterial community, which is directly tied to soil nutrient recycling (Jacoby et al., 2017). In this regard, it has been reported that the abundances of Proteobacteria and Acidobacteria are related to the nutrient status of soils, and high Proteobacteria/Acidobacteria ratios are indicative of copiotrophic soils (Smit et al., 2001; Babujia et al., 2016; Huang et al., 2020). This could be partially explained or supported by that the Proteobacteria is responsible for several biogeochemical functions in the soil, such as symbiotic N fixation and nutrient cycling remineralization, while the Acidobacteria has a propensity to thrive in oligotrophic conditions, typically coupled with lower plant productivity (Lewis et al., 2012). In this study, biochar application resulted in an increase in the relative abundance of Proteobacteria but a decrease in the relative abundance of Acidobacteria. This means that a higher Proteobacteria/Acidobacteria ratio was induced by biochar application, suggesting that biochar application improved the soil nutrient status. This might also be partially responsible for the improvements in root length, surface area, and dry weight that resulted from biochar application.

**Figure 8:**
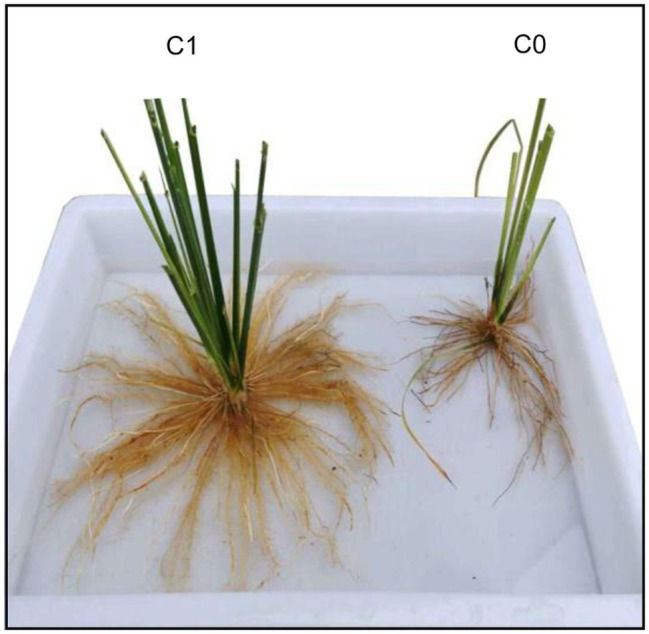
Comparison on roots of rice plants grown with biochar application (C1) and without biochar application (C0).

In contrast to the effect of biochar application on increasing root length, surface area, and dry weight in rice plants, we found that root diameter was reduced in the biochar treatment (C1). This was because biochar application facilitated the growth of many more lateral roots with smaller diameter in the rice plants (Figure 8). This observation indicates that biochar application improved root architecture because the lateral roots are critical to allowing the plants to take up water and minerals effectively (von Wangenheim et al., 2020). Auxin is required for lateral root formation. In the present study, biochar application led to a down-modulation of an auxin efflux carrier component (OsI_23989) in roots, leading to the accumulation of auxin in lateral root initials and promotion of lateral root growth (Kazan, 2013). Moreover, it has been documented that increasing endogenous abscisic acid and ethylene can inhibit lateral root formation in plants (Negi, 2008; Guo et al., 2009). In this study, ASR5 induced by abscisic acid (Jia et al., 2016) and ethylene-insensitive 2 (OsI_24945) involved in ethylene biosynthesis (Singh et al., 2015) were down-modulated in roots by biochar application. This indicates that reductions in abscisic acid and ethylene biosynthesis could also be responsible for the increased number of lateral roots in the biochar treatment.

Nitrate acts as a crucial signal in the regulation of lateral root development, and nitrate transporters have negative effects on lateral root development under low nitrate conditions (Sun et al., 2017). In this study, (1) the experiment was conducted under flooding conditions, where the soil nitrate should be low because nitrate replaces oxygen as the terminal electron acceptor in microbial respiration leading to denitrification and/or nitrate ammonification under flooding (Laanbroek, 1990); and (2) the application of biochar resulted in down-modulation of two nitrate transporters (OsI_13072 and OsI_27853). These results show that the increased number of lateral roots in the biochar treatment could also be related to the repression of nitrate transporters.

In addition to improve the morphological and architectural traits, biochar application up-modulated glutamine synthetase (OsI_08842 and OsI_10575), glutamine amidotransferase type-2 domain-containing protein (OsI_03285), and two ammonium transporters (OsI_08109 and OsI_16598) in the root. As a consequence, shoot N uptake in rice plants was increased by biochar application. It is well known that N plays an important role in improving photosynthesis and growth in rice (Yin et al., 2017). Consistently, in this study, leaf area, SLW, LNC, most differentially modulated proteins related to photosystems I and II, tiller number, and shoot dry weight in rice plants were increased or up-modulated in parallel with increased N uptake due to biochar application. These improvements in shoot traits could be in turn responsible for the improved root morphological, architectural, and physiological traits resulting from biochar application, because root establishment and maintenance require assimilates produced by the shoot (Yang et al., 2012). Furthermore, the synchronous improvements in root and shoot traits could accelerate the transport of water in the soil-plant-atmosphere system, might resulting in transpiration cooling, and helping plants to avoid heat stress damage (Jagadish et al., 2015). However, additional studies are required to confirm this potential mechanism.

**Figure 9:**
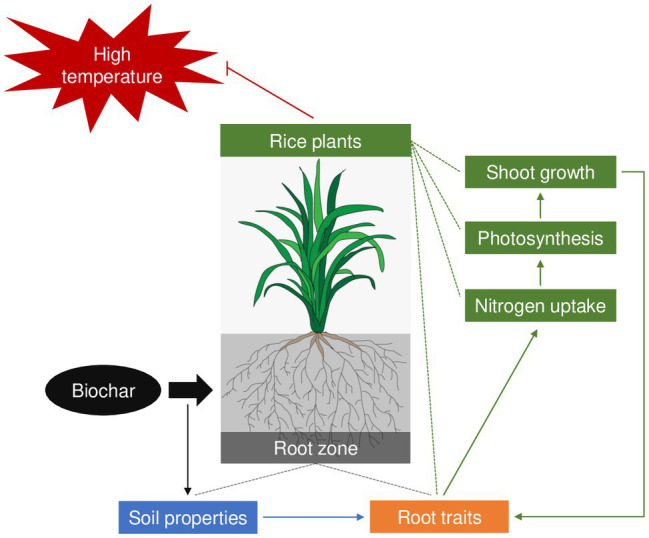
A schematic diagram for the eco-physiological processes underlying the effects of biochar on resistance to heat stress in rice plants.

Taken together, the results of this study suggest that the application of biochar can improve soil physiological, chemical, and biological properties and consequently improve root morphological, architectural, and physiological traits as well as shoot N uptake and utilization, which ultimately mitigate the effect of heat stress on rice plants (Figure 9). This finding not only increases our understanding of the fundamental eco-physiological processes underlying increased heat-stress tolerance in rice plants that results from biochar application, but also implies that improving the root-zone environment by optimizing management practices is an effective strategy to mitigate heat stress in rice production. The finding of this study also highlights the need for further investigations to compare the effects of biochar application on the growth and eco-physiological characteristics in rice plants between normal- and high-temperature conditions.

## Data Availability Statement

The original contributions presented in the study are publicly available. This data can be found at: https://figshare.com/articles/dataset/MS_identified_information_xlsx/14945382/1.

## Author Contributions

MH conceived the experiment, analyzed the data, and wrote the manuscript. XY, JC, and FC performed the experiment. All authors have read and approved the final manuscript.

## Conflict of Interest

The authors declare that the research was conducted in the absence of any commercial or financial relationships that could be construed as a potential conflict of interest.

## Publisher’s Note

All claims expressed in this article are solely those of the authors and do not necessarily represent those of their affiliated organizations, or those of the publisher, the editors and the reviewers. Any product that may be evaluated in this article, or claim that may be made by its manufacturer, is not guaranteed or endorsed by the publisher.
